# Nutrition, Physical Activity, and New Technology Programs on Obesity Prevention in Primary Education: A Systematic Review

**DOI:** 10.3390/ijerph181910187

**Published:** 2021-09-28

**Authors:** Lorenzo Navidad, Rosario Padial-Ruz, Mar Cepero González

**Affiliations:** Department of Didactics of Musical, Plastic and Corporal, University of Granada, 18011 Granada, Spain; lorenbetula@correo.ugr.es (L.N.); mcepero@ugr.es (M.C.G.)

**Keywords:** intervention, nutrition, health, exercise, ICT, primary stage

## Abstract

Early acquisition of healthy lifestyle habits is crucial for good adult health. For this reason, the primary stage of education is a critical period to implement educational policies in this regard. The aim of this review is to compile the published evidence regarding school interventions at the primary stage aimed at preventing obesity, and which integrate as part of their action plan two features: an improvement in knowledge or nutrition habits and the promotion of physical activity (PA), and the use of new information and communications technologies (ICT) to do this. The method used for this review is the searching of different databases for publications that include these criteria. The results show beneficial effects of such interventions in improved eating habits and increased PA. The effect on BMI is limited, and the use of ICT can be of help at a motivational level for the maintenance and fulfilment of the health objectives. However, studies of this type in elementary school are very limited, so it would be necessary to continue researching on this line. In conclusion, this review demonstrates the suitability of carrying out mixed interventions (improved nutrition and PA) together with the use of new technologies to improve health and prevent obesity at an early age.

## 1. Introduction

The prevalence of obesity in children and young people has reached dramatic dimensions worldwide and remains one of the most challenging problems in developed countries [1]. Ten percent of the world’s school-aged children are estimated to be carrying excess body fat. Of these overweight children, a quarter are obese [2]. This is a problem not least because children who are overweight or obese are more likely than those who are of normal weight to experience other significant health problems in childhood [3].

Schoolchildren are at a much higher risk of being overweight and obese if they follow a sedentary lifestyle and do not take part in sport or other physical activities outside school [4]. Therefore, the level of PA is an important determinant in the prevention and treatment of childhood obesity and early metabolic risk factors [5]. However, children showed a persistent global trend toward low PA and high sedentary behaviors [6]. Globally, it is estimated that only one fifth of young people are physically active enough [7]. Although the development of childhood obesity is multifactorial, decreased energy expenditure is considered one of the most important determinants of excess body weight [8]. PA is the most modifiable factor in energy expenditure, it represents approximately 25% of total expenditure and, as such, is a powerful lever to improve the energy balance equation [9]. This is why having a high level of PA is associated with a lower BMI and measured body fat, even after controlling for genetic factors and the childhood environment [10]. Active play (unstructured, outdoor PA in children’s free time), cycling, or walking instead of travel by car or bus, and participation in sports are the main contributors to the total PA load among children [11]. At least 60 min a day of moderate to vigorous physical exercise is recommended for schoolchildren in the primary age-group [12]. However, the proportion of children who reach these levels is very low, especially with respect to girls [13,14]. As if that were not enough, we know that the time spent doing PA decreases as children grow older [15,16]. This situation could be improved by early intervention, since various investigations have shown that healthy PA habits established during childhood can be maintained into adulthood [17].

Traditional nutritious foods, which tend to be high in complex carbohydrates and vegetables, are being replaced by foods high in fat and calories [18]. Currently, ultra-processed food products contribute a large part of the calories in the children’s diet [19,20,21]. The consumption of processed foods with a high content of fats and refined sugars is gaining prominence to the detriment of those that constitute the pillars of a healthy diet, such as fruit, vegetables, legumes, cereals, or fish [22]. We know that the high intake of processed foods and the low intake of fruits and vegetables are key factors in the development of childhood overweight and obesity [23]. In addition, such a diet is associated with a worse cardiometabolic risk profile, an increased risk of cardiovascular disease, cerebrovascular disease, depression, and mortality [24]. To prevent this, it is necessary to increase the intake of healthy foods and reduce the intake of unhealthy foods [25]. Regarding the reduction of unhealthy foods, although it may be thought that the most dangerous products are sweets, these do not usually play a significant role since they are usually consumed on an occasional and irregular basis [26,27]. Regarding the increase in healthy foods, it has been observed that the incorporation of fruits and vegetables into schoolchildren’s meals can reduce the total intake of calories by reducing energy density [28], in addition to being associated with a decrease in the consumption of unhealthy foods [29,30]. Be that as it may, these healthy eating habits should be promoted from infancy, since the eating patterns established in early childhood tend to persist into later ages [31].

As technologies have advanced, so has the development of new tools to measure diet and PA [32]. Regarding their use for obesity prevention, they have the potential to make more effective interventions in health behaviour [31,33,34]. In fact, digital interventions are becoming increasingly popular in effecting lifestyle changes. These interventions show the best results when combined with counselling and feedback [31]. For example, tools using forms of communication such as mobile phones, SMS, and so on are very useful because they allow feedback to be provided in real time and at a lower cost than sending people to school [35,36]. Web platforms or applications are also a potentially promising tool because more and more people have access to the Internet, and it has been shown that an intervention based on the use of websites is at least as effective as traditional methods [37]. Technologies such as video games are proving to be an additional complementary intervention strategy offering attractive methods to attract attention, educate, and promote behavioural changes [31]; they may even have positive effects on knowledge of nutrition, dietary, and PA behaviours in schoolchildren [31,38]. Therefore, there is a need to better understand how human movement culture and school physical education are co-evolving alongside the development of new media technologies [39].

Children spend at least a third of their waking time in schools, and therefore interventions for the prevention of obesity should always include the school as well as the family and community in order to achieve long-term effects on children’s health [40]. Regarding families, they play a fundamental role in the prevention of obesity. Some studies find that prevention is more effective if starts at an early age and if it involves families [31,41].

Previous reviews have had the objective of evaluating existing interventions for the prevention of over-weight and obesity, but none of these reviews has focused on the prevention of obesity in the primary stage through an improvement of eating behaviours and taking part in PA and using new technologies to do so. Thus, the aim of the present study was to carry out a systematic review focusing on the identification of the general characteristics and the effectiveness of the intervention programmes carried out during primary schooling.

## 2. Materials and Methods

The review was performed following the PRISMA 2020 statement: an updated guideline for reporting systematic reviews [42]. 

### 2.1. Search Strategy

A comprehensive search of five electronic databases: ProQuest, PubMed, Scopus, SPORTDiscus, and Web of Science during the first weeks of June 2021.

Specifically, all databases were considered, the time range was limited to studies published from 2010 onwards and the type of document was limited to articles. The query string was: “childhood obesity” AND intervention AND nutrition AND (“physical activity” OR exercise) AND (technology OR ICT OR digital OR “serious games” OR mobile OR web OR app OR sms OR mhealth).

### 2.2. Inclusion Criteria

Studies were eligible if they: (1) were published in Spanish or English and submitted to peer review; (2) were aimed at primary schoolchildren; (3) carried out an intervention to improve eating habits or the taking part in PA; (4) used new technologies to carry out said intervention; (5) and showed results and conclusions.

### 2.3. Data Screening

All search results were exported to the Zotero library and duplicates removed. The title and abstract of the retrieved articles were selected, using the inclusion criteria described above, by one reviewer and verified by another. If a study was mentioned multiple times, only the most recent publication was included in the analysis. The reference lists of studies included, and related systematic reviews, were examined to identify any additional studies. The full text of the remaining articles was then reviewed to determine final inclusion. Discrepancies in study inclusion were resolved by discussion with a third reviewer.

### 2.4. Data Extraction

The categorization and analysis were carried out with the help of the ATLAS.to software (version 9, Scientific Software Development GmbH, Berlin, Germany). One reviewer extracted the data and another checked its accuracy. From each study included, the following characteristics were extracted: data source, population characteristics, sample size, study design, duration of the intervention, intervention performed, measured variables, instruments used for data extraction, new technology used, results and main conclusions of the study. The quality of the studies was quantitatively analysed using descriptive statistics (absolute frequency).

### 2.5. Assessment of the Methodological Quality of the Studies

The risk of bias in each eligible article was assessed by adopting a dichotomous nominal scale of two unique values (yes/no), which was developed to assess concordance in the 14 studies in the sample. As variables of the scale, the criteria are indicated in Section 2.2 (Inclusion Criteria). The degree of agreement obtained in the classification of the works was 93%, which was obtained by dividing the number of coincidences by the total number of categories defined for each study and multiplying it by 100.

## 3. Results

### 3.1. Database Searches

**Figure 1:**
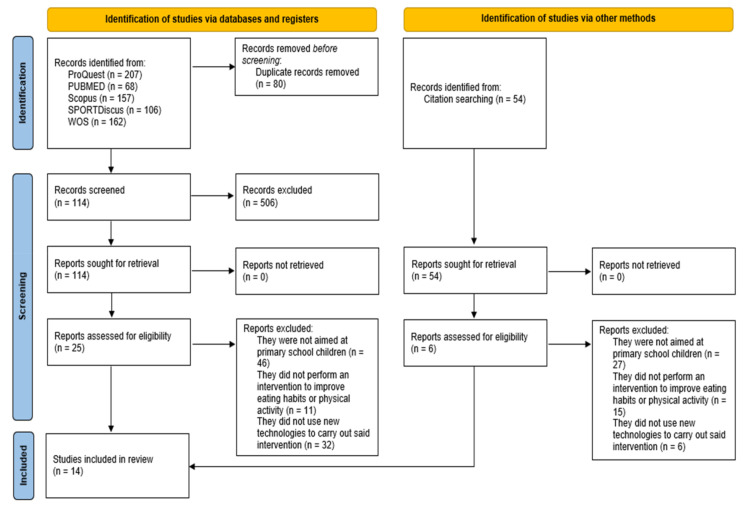
PRISMA 2020 flowchart of the systematic review inclusion process.

A PRISMA flow chart in Figure 1 illustrates the identification, selection, eligibility, and inclusion of studies within the systematic review. The database search yielded 620 articles. A total of eight articles met the inclusion criteria, while an additional six articles were retrieved after reviewing the reference lists of included studies and other systematic reviews. In total, 14 studies were eligible for inclusion in the systematic review.

### 3.2. Description of Included Studies

The characteristics of the included studies are described in Table 1. 

The number of children who participated in the study ranged from 60 participants [52] to 4846 participants [54]. The age of the participants in each study ranged from 4 years [50] to 13 years [49,54]. Interventions were heterogeneous with respect to study duration, type of interventions, and outcome measures. The duration of the interventions ranged from 9 lessons [55] to 4 years [48], 29% of the interventions (*n* = 4) had a duration of less than 1 month, 29% of the interventions (*n* = 4) had a duration of between 1 month and 3 months, 14% of the interventions (*n* = 2) had a duration of between 3 months and 2 years, and 29% of the interventions lasted more than 2 years (*n* = 4).

Below (Table 2), a summary of the results, chronologically, and a conclusion of each study in the sample is presented.

### 3.3. Effects of Interventions on Nutrition

Although all the studies intervened in aspects of improving knowledge and/or eating habits, Grydeland et al. [45] did not measure whether there was a change in this aspect. Shamah-Levy et al. [43] found statistically significant differences between intervention and control groups with regard to knowledge about eating (*p* = 0.000). Williamson et al. [44] found no difference for changes in food intake, although they did in fat consumption (F = 4.86, *p* = 0.04). Burke et al. [46] observed significant improvements over time in student health-related knowledge, self-efficacy, and behaviors (*p* < 0.0001). Fassnacht et al. [47] also found significant differences in favour of the intervention group. Silva et al. [48] found significant changes in the intake of fruit and vegetables (B = 0.97, *p* < 0.05). Grutzmacher et al. [49] found that between 23% and 35% of parents reported an improvement in nutrition. Jungwon Min et al. [50] reported that the change in children’s eating habits was marginally significant. Bartelink et al. [51] reported that healthy eating behaviours and lunch intake improved significantly more in the intervention group. Wadolowska et al. [52] observed a greater increase in the nutritional knowledge score (1.8 pts.) but no significant difference in terms of changes in the quality of the diet. Espinosa-Curiel et al. [53] found greater knowledge about eating, improving from 56.95 to 67.88 out of 90 total points, an increase in the consumption of healthy food from 1.5 to 2.25 and a decrease in unhealthy food from 2.35 to 1.25 (0 = never, 1 = once or twice a month, 2 = three or more times a month, 3 = once or twice a week). Mack et al. [54] reported a significant increase in nutritional knowledge in the experimental group, but there were no significant changes between groups in terms of dietary behaviour. Xu et al. [55] did not find improvements in dietary diversity and the variety of foods in general (effect = 0) but did find improvements in the variety of foods consumed at breakfast (effect = 0.1) and in an increase of the consumption of cereals and fruit (effect 1.4). Finally, Sánchez-Martínez et al. [56] found non-significant positive changes in the consumption of water, meat, sweets and chips, but did not find significant differences in the global nutrition score between the experimental group (44.3%) and the control group (41.1%).

### 3.4. Effects of Interventions on Physical Activity

Although all the study interventions were positive in aspects of improvement of PA knowledge and/or habits, Grutzmacher et al. [49], Espinosa-Curiel et al. [53], and Xu et al. [55] did not measure whether there was a change in this aspect. Shamah-Levy et al. [43] found statistically significant differences between intervention and control groups with regard to knowledge about physical activity (*p* = 0.028). Williamson et al. [44] found no differences for changes in total daily PA. Grydeland et al. [45] observed an effect on the total PA at the level of *p* = 0.05 in favour of the intervention group. Burke et al. [46] observed improvements in the amount of Physical Education and PA. Fassnacht et al. [47] found significant differences in favour of the intervention group. Jungwon Min et al. [50] reported that the behaviour in PA improved significantly. Bartelink et al. [51] observed that the percentage of sedentary time had decreased and the percentage of time spent in light PA had increased more in the intervention group. Wadolowska et al. [52] observed that the probability of adherence to the WHO recommendation on PA was significantly higher at 74%, although they also found a decrease in PA in the experimental group. Finally, Sánchez-Martínez et al. [56] found a non-significant increase in PA outside school and a significant difference in the global activity score between the experimental group and the control group. On the other hand, Silva et al. [48] and Mack et al. [54] did not find significant differences in terms of total daily PA.

### 3.5. Effects of Interventions on Body Mass Index

Although all the studies intervened with the aim of preventing obesity, not all measured whether there was a change in BMI. Shamah-Levy et al. [43] found that their intervention was effective in maintaining BMI, but not in reducing it because the intensity and duration of the programme’s PA were not sufficient to have a notable effect on BMI, and other important variables were not controlled for. Williamson et al. [44] did not find significant changes in terms of BMI (♀ F = 2.68, ♂ F = 2.47), although there was an improvement close to statistical significance in the percentage of body fat (F = 4.26); neither did Burke et al. [46], in the BMIz, nor Silva et al. [48] in the IMC-SDS. Bartelink et al. [51] found a small but significant decrease in BMI. Wadolowska et al. [52] observed a greater decrease in z-WHtR and waist circumference z. Finally, Sánchez-Martínez et al. [56] found no differences in BMI between groups.

### 3.6. Effects of the Use of New Technologies

Only five studies mentioned the effect of the use of new technologies in the intervention. Williamson et al. [44] found that, with regard to the prevention of weight/fat gain, there was not an improvement, but there was in PA maintenance, and in the support of teachers in dietary changes and social support in overweight students. This suggests that the Internet programme may be more applicable in interventions that emphasize changes in PA and in improving social support. Fassnacht et al., Silva et al., and Mack et al. [47,48,54] observed that the students were satisfied with the use of the programme, with the use of SMS and the use of the pedometer. Finally, Espinosa-Curiel et al. [53] concluded that most families agree that, when playing video games, their sons and daughters showed greater interest in various healthy eating behaviours.

## 4. Discussion

This review synthesized the evidence for the efficacy of school interventions in preventing obesity among primary schoolchildren.

Regarding the improvements found in knowledge about food and food intake, these could be due to the use of the nutrition-education approach in the tasks carried out in the programmes [56], or to the appropriate duration of the intervention programme [43]. In the case of Burke et al. [46], this improvement was higher in the first two years than in the third. This may be due to the possible existence of a threshold beyond which achieving an improvement is more difficult to accomplish.

Regarding the improvements found in the knowledge of and involvement in PA, these could be due to the performance of increased PA activities in the tasks carried out in the programmes [56], or the duration of the PA programme intervention that was within adequate ranges [43]. In the case of Burke et al. [46], this improvement was higher in the first two years than in the third. This can be said to a possible threshold beyond which achieving an improvement is more difficult to bring about, or to an insufficient sample size. In studies where general PA was increased, this could be due to the fact that special emphasis was placed on promoting PA, rather than on high-intensity activities, or also due to seasonal variation in measurements, since the initial measurement was made in autumn and the later one in spring [45]. In the studies where a general increase in PA was not shown, this could be due to the short duration of the intervention [54] because the sample already met the daily recommendations before the intervention, so they were already physically active [48], or it could be that a general reduction in PA is inevitable with increasing age [52].

Regarding the improvements or maintenance of the BMI found, these could be due to the fact that the interventions that combine healthy eating habits and PA are generally effective in this regard [43,52]. As for the studies that do not observe significant differences in BMI, this could be due to the fact that the interventions are aimed more at a change in behaviour that lays the foundations for good health-lifestyle habits. Therefore, the change in BMI would be a later consequence of these changes in habits and, in order to observe them, the intervention time should be increased [44].

Regarding the improvements shown by the use of new technologies, these could be due to the fact that they allow personalized feedback that favours the influence on the change in health behaviour and its maintenance [44,48], to the high adherence of children to improved technology systems [47], or to the high level of challenging interactions, repetitions and the self-reflection tools applied in play [54]. Regarding the case where no improvement was observed in the prevention of weight gain, when using the programme based on new technologies, this could be due to an incorrect implementation of it by the teachers, a suboptimal participation by the families, or to an insufficient duration of the intervention or the influence of summer holidays [44].

## 5. Strengths and Limitations

This review contributes to the existing evidence base; to the extent of our knowledge, it is the first systematic review of school interventions for obesity-prevention focused on diet and PA using new technologies in primary schoolchildren. The findings should be interpreted with caution considering the following limitations. First, the high level of heterogeneity detected in the included studies, which is a common finding among multi-component obesity interventions, limits the robustness of these findings. Second, in most cases, self-reported questionnaires were used, which are always open to information bias, a difficulty in clearly remembering previous experiences or the exaggeration/underestimation of the information reported. Third, the scarcity of studies that include the use of new technologies makes it impossible to discern which of these are the most interesting to use, or make a review exclusively of a specific technology. Finally, some interventions had a short period of time of application, so that only short-term results could be observed and without being able to verify the possible maintenance of the same, or potential long-term benefits.

## 6. Conclusions

The findings of our review should be considered with caution due to the great heterogeneity of the sample. It seems that the efficacy of interventions in obesity prevention is generally positive. Although the most common intervention in PA is by increasing its time, and in nutrition by food education, the best approach to achieve significant differences in both cases is not clear. The effect on BMI of the interventions is limited. Regarding the use of new technologies, positive results are shown in changes in behaviour and in the acquisition of improved habits, although it is not clear what type of new technology is better to use.

Future research should consider performing such interventions in the general population and not to focus only on children at risk of overweight/obesity or who already suffered from it. They should at least have a control group and try to evaluate specifically if the use of new technology was positive.

We believe that the practical application of this review focuses on its usefulness for primary schools that want to prevent obesity in their centres. Regarding its didactic implications, we highlight the importance of giving families greater prominence so that they feel part of the proposed change to improve health, and the taking into account of the school context, which has been shown to have special importance for the acquisition of healthy habits.

## Figures and Tables

**Table 1:** Analysis of intervention programmes.

Study	Sample	Design	Length	Intervention	Variables	Instruments	ICT
Shamah-Levy et al. (2012) [43]	1020 (♀ 50.7%, ♂ 49.3%) (10–11 years old)	Blind cluster-randomized field trial	3 weeks (6 months follow-up)	Students: Nutrition and PA workshops, creation of puppet theatre, activation sessions and active participation games at playtime breaks. Teachers: Workshops to raise awareness about healthy eating and PA. Families: Calendars with healthy school breakfast recipes. School: Training of workers to recommend vegetables, fruit and water, delivery of water bottles, banners and public address announcements to promote the consumption of fruits, vegetables, water and PA.	BMI; Food intake; PA KDPA; PAS; HES	ES + S; FFQ; PAQ; MCQ; DCQ	Video
Williamson et al. (2012) [44]	2060 (♀ 58.5%, ♂ 41.5%) (9–12 years old)	Longitudinal, cluster randomized 3-arm controlled	28 months	Primary Prevention (PP): Environmental modification programme to promote healthy diet, PA and prevention programme for families. Primary + Secondary Prevention (PP + SP): Primary prevention + Classroom curriculum + counselling and education on the Internet.	Body fat; BMIz; FI; PA SST	IS + S; Digital video camera; SAPAC; DSS	Website
Grydeland et al. (2013) [45]	700 (♀ 57%, ♂ 43%) (11 years old)	Cluster randomized controlled trial	20 months	Students: Lessons on nutrition, consumption of fruits and vegetables, active breaks in the classroom, active transport campaigns, pedometers and computer-tailored individual advice. Families: Information sheets about nutrition and PA. School: Teacher training in the SPARK Physical Education teacher-training program.	BMI; A	ES + S; PCS; ActiGraph; 7164/GT1	Computer tailored programme
Burke et al. (2014) [46]	40 schools (8–11 years old)	Pretest-post-test	3 years	The programme is integrated into the curriculum to improve health, PA and nutrition-education knowledge and behaviours. In addition, there are classroom exercises with the DVD, assemblies, classroom lessons, and family-based reinforcement activities.	HKB; BMI; Cardiovascular fitness; SSLP	Ad-hoc Questionnaire ES + S; PACER CITT	Video
Fassnacht et al. (2015) [47]	49 (♀ 53%, ♂ 47%) (8–10 years old)	Pretest-post-test	8 weeks.	All children participated in 2 educational group sessions that focused on health behaviours. The monitoring group also reported daily behaviours using SMS and received supportive feedback.	BMIz; Health behaviour; Daily fruit & vegetable intake; PA	ES + S; Questionnaire; Pedometer	SMS
Silva et al. (2015) [48]	139 (♀ 47.5%, ♂ 52.5%) (8–10 years old)	Pretest-post-test	8 weeks (4 weeks follow-up)	Two educational sessions of nutrition, PA, and screen-time and monitoring of the experimental group by SMS.	FVI; PA + CT; Daily steps; BMI; SP	FFQ; FEAHQ; Pedometer Plus;ES + S; SSQ	SMS
Grutzmacher et al. (2018) [49]	23 schools	Pretest-post-test	4 years	Text2BHealthy schools and control schools received standard classroom-based nutrition education from FSNE educators and classroom teachers trained by FSNE educators. Parents from Text2BHealthy schools receive 2 text messages each week during the school year and 2–3 messages each month during the summer.	FPB; HNE; DC; CBRE; PAB; ST	Ad-hoc Survey	SMS
Jungwon Min et al. (2018) [50]	409 (♀ 52.3%, ♂ 47.7%) (6–13 years old)	Pretest-post-test	6 weeks	Activity from website was completed in about 30 to 50 min per class time. The participants could repeat the activities with the teacher 2 or more times within a week.	Obesity-related behaviours; Health knowledge	Questionnaire	Website
Bartelink et al. (2019) [51]	1676 (♀ 52.6%, ♂ 47.4%) (4–12 years old)	Longitudinal quasi-experimental	2 years	Partial HPSF (PA): E-health programme for parents, structured PA sessions after lunch. Full HPSF (PA + Nutrition): Partial HPSF + Improved their health policy, provided water bottles and provided an educational lunch once a week.	BMIz; Socioeconomic status; Children’s; PA; behaviours Children’s dietary behaviours Children’s lunch intake	ES + S; Parent questionnaire; Accelerometer; Child questionnaire; Child lunch questionnaire	E-health program
Wadolowska et al. (2019) [52]	646 (♀ 53.4%, ♂ 46.6%) (11–12 years old)	Pretest-posttest	3 weeks (9 months follow-up)	The programme consisted of 5 topics, each topic included various forms of education from fun to “scientific” cognition. Each topic lasted approx. 180 min (4 h of school lessons) and was run by a minimum of 3–4 researchers.	Diet, sedentary and active lifestyle; Nutrition knowledge Sociodemographic characteristic	Short Form of the Food; Frequency; Questionnaire for Polish Children	Website
Espinosa-Curiel et al. (2020) [53]	60 (♀ 47%, ♂ 53%) (8–10 years old)	Pretest-post-test	6 weeks	12 sessions of at least 25 min of play.	Food knowledge Dietary intake Parent perception	FKQ; FFQa; PPQ	Serious video game
Mack et al. (2020) [54]	82 (♀ 48%, ♂ 52%) (9–12 years old)	Cluster randomized controlled trial	2 weeks (4 weeks follow-up)	Intervention group: played the game (45 min) twice over a 2-week period, with a different selection of game modules played at each of the two sessions. Control group: Received basic information about a healthy lifestyle via a brochure.	Maintenance of knowledge Acceptance of the game; Emotions during game play Changes in dietary behaviour; PA; Media consumption	Knowledge questionnaire Questionnaire Self-assessment manikin Ernährungsmusterindex; KIGGS	Serious video game
Xu et al. (2020) [55]	4846 (7–13 years old)	Randomized controlled trial	12 months	Nutrition-education intervention (NE): Food notebook, courses for students (6), parents (2) and teachers (4), poster and class meetings. PA intervention (PA): Course for parents, 20 min of “Happy 10” per day and class meetings. Mixed group (CNP): All of the above.	BMI; DC; DDS9; DDS28; FVS	ES + S; Parent questionnaire; 24hDR	Video
Sánchez-Martínez et al. (2021) [56]	4139 (8–9 years old)	Pretest-posttest	9 lessons of 55 min. Reinforcement: 2 lessons of 50 min. and 1 of 60 min. (1 and 3 years follow-up)	Individual: Class on nutrition and registration of PA in 1 month. Family: Workshops to improve food and PA on weekends and attendance at more than 4 events at weekends. School: Review of the school menu, improvement in the availability of healthy food in the cafeteria and promotion of the opening of spaces.	BMI; TST; Physical fitness	P-HDWE; P-HDWM; Eurofit battery	Digital platform “Edu Natura”

**Note 1:** Body mass index (BMI); Knowledge about diet and physical activity (KDPA); Physical activity self-efficacy (PAS); Healthy eating self-efficacy (HES); Body Mass Index z scores (BMIz); Food intake (FI); Social Support from Teachers (SST); Health knowledge and behaviour (HKB); Self-assessment of school-level progress (SSLP); Fruit and vegetable intake (FVI); Physical activity and screen time (PA + CT); satisfaction with the programme (SP); Food Purchasing Behaviours (FPB); Home Nutrition Environment (HNE); Demographic Characteristics (DC); Children’s Behaviour Related to Eating (CBRE); Physical Activity Behaviours (PAB); Screen Time (ST); Triceps skin-fold thickness (TST); Dietary Diversity Score for 9 food groupings (DDS9); Dietary Diversity Score for 28 food groupings (DDS28); Food Variety Score (FVS). **Note 2:** Electronic scale and stadiometer (ES + S); Food Frequency Questionnaire (FFQ) [57]; Physical activity questionnaire (PAQ) [58]; Multiple-choice questionnaire (MCQ); Dichotomous-choice questionnaire (DCQ); Impedance scale and stadiometer (ES + S); Self-Administered Physical Activity Checklist (SAPAC); Children’s Dietary Social Support scale (DSS) [59]; Pubertal Category Scores (PCS) [60]; Progressive Aerobic Cardiovascular Endurance Run (PACER) [61]; Continuous Improvement Tracking Tool (CITT); Family Eating and Activity Habits Questionnaire (FEAHQ) [62]; Self-report satisfaction questionnaire (SSQ); POIBA-How do we eat? (P-HDWE); POIBA-How do we move? (P-HDWM); Food Knowledge Questionnaire (FKQ); Adapted Food Frequency Questionnaire (FFQa); Parent Perception Questionnaire (PPQ); German Health Interview and Examination Survey for Children and Adolescents (KIGGS) [63]; 24-h dietary recall (24hDR).

**Table 2:** Results and conclusions of the interventions.

Study	Results				Conclusion
	N	PA	BMI	ICT	
Shamah-Levy et al. (2012) [38]	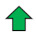	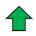	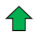	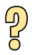	The intervention strategy is effective in maintaining the BMI of schoolchildren.
Williamson et al. (2012) [39]	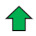	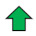	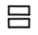	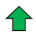	This school-based environmental-modification programme had modest beneficial effects on changes in percentage of body fat. Addition of a classroom/internet programme to the environmental programme did not enhance weight/fat-gain prevention, but did enhance physical activity and social support in overweight children.
Grydeland et al. (2013) [40]	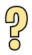	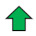	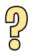	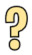	An implementation of the HEIA intervention components in the school system may have a beneficial effect on public health by increasing overall physical activity among adolescents and possibly among girls and low-active adolescents in particular.
Burke et al. (2014) [41]	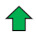	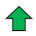	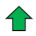	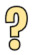	The HealthMPowers programme is effective in producing positive change in school policies and practices, student knowledge and behaviours, and student fitness and BMI, supporting the use of holistic interventions to address childhood obesity.
Fassnacht et al. (2015) [42]	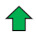	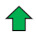	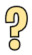	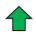	The current SMS intervention was a useful tool to monitor and promote improved health behaviours in children.
Silva et al. (2015) [43]	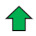	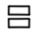	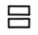	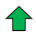	The present findings suggest that the SMS-based monitoring and feedback systems have the potential for promoting better health behaviours in children.
Grutzmacher et al. (2018) [44]	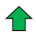	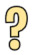	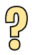	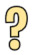	Text2BHealthy resulted in improvements in a number of fruit and vegetable -consumption practices of parents and their children.
Jungwon Min et al. (2018) [45]	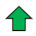	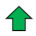	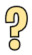	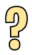	NASA MX programme was shown to improve children’s health knowledge and PA in the United States.
Bartelink et al. (2019) [46]	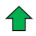	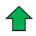	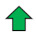	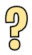	Full HPSF is effective in promoting positive health behaviours in children at T1 and T2 compared with control schools. Focusing on both nutrition and PA components seems to be more effective in promoting healthy behaviours than focusing exclusively on PA.
Wadolowska et al. (2019) [47]	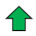	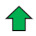	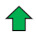	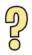	In conclusion, diet-related and lifestyle-related school-based education from an almost one-year perspective can reduce central adiposity in pre-teenagers, despite a decrease in physical activity and the tendency to increase screen time.
Espinosa-Curiel et al. (2020) [48]	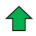	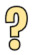	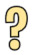	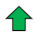	Health games such as FoodRateMaster are viable tools to help young children increase their food knowledge and improve dietary behaviours.
Mack et al. (2020) [49]	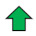	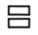	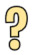	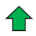	The Kids Obesity Prevention programme sustainably increased knowledge in the areas of nutrition and coping with stress, and children were able to apply the dietary energy density principle (DED-P).
Xu et al. (2020) [50]	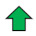	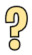	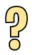	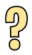	Though the comprehensive obesity intervention did not improve the overall dietary diversity per day, positive intervention effects were observed in the consumption of breakfast and some other foods.
Sánchez-Martínez et al. (2021) [51]	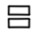	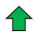	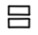	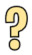	School-based interventions are a good strategy to tackle the global rise in childhood obesity. Multilevel and multicomponent school-based interventions, including a family component, could improve children’s health habits, especially those regarding food and nutrition, and the taking part in physical activity. They could also be helpful in preventing the appearance of new cases of childhood obesity, though they may not have an immediate effect on adiposity outcomes.

**Note 1:** Nutrition (N), Physical activity (PA), Body mass index (BMI), Information and Communication Technologies (ICT). **Note 2:** Significant improvement ( 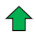), non-significant difference ( 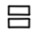), Not measured for outcomes ( 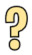).

## Data Availability

Data are contained within the article.
